# Archaea in Natural and Impacted Brazilian Environments

**DOI:** 10.1155/2016/1259608

**Published:** 2016-10-17

**Authors:** Thiago Rodrigues, Aline Belmok, Elisa Catão, Cynthia Maria Kyaw

**Affiliations:** Department of Cell Biology, Biological Sciences Institute, University of Brasília, 70910-900 Brasília, DF, Brazil

## Abstract

In recent years, archaeal diversity surveys have received increasing attention. Brazil is a country known for its natural diversity and variety of biomes, which makes it an interesting sampling site for such studies. However, archaeal communities in natural and impacted Brazilian environments have only recently been investigated. In this review, based on a search on the PubMed database on the last week of April 2016, we present and discuss the results obtained in the 51 studies retrieved, focusing on archaeal communities in water, sediments, and soils of different Brazilian environments. We concluded that, in spite of its vast territory and biomes, the number of publications focusing on archaeal detection and/or characterization in Brazil is still incipient, indicating that these environments still represent a great potential to be explored.

## 1. Introduction

**Figure 1:**
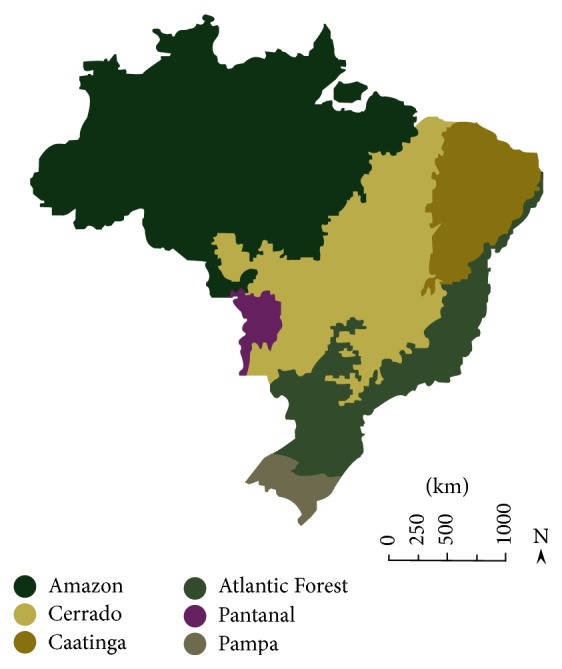
Schematic representation of Brazilian biomes distribution along the country's territory.

Brazil is a country widely known for its continental proportions and biodiversity. It is the largest country in South America and the fifth largest in the world, with its territory covering an area of 8,514,876.599 km^2^ [1]. Because of the country's great range of latitudes, there are many different terrestrial landscapes and biomes within Brazil (Figure 1).

The Amazon biome, located in the north of Brazil, corresponds to 49.29% of the country's territory and is mainly constituted by tropical forests, which are notable for harboring a diverse flora and fauna [2]. Cerrado, also known as the Brazilian savanna, corresponds to the second largest biome in Brazil and is located in the central part of the country. It has varied phytophysiognomies, ranging from grasslands to forests [3], and two defined seasons—dry and wet—and is also characterized by the occurrence of natural fires [4]. The Atlantic Forest extends along the Atlantic coast of Brazil and has different ecological regions within the biome. It is known by the presence of a high diversity of species, many of which are endemic [5]. The Caatinga biome is distributed on the northeastern portion of the country, with a desert vegetation, consisting in xeric shrublands and thorn forests. Like Cerrado, it also has a seasonality regimen based on the occurrence of wet and dry periods [6]. Pampa, located in the south region, is characterized by a typical vegetation of grassland, sparse shrubs, and tree formations [7]. Pantanal is the world's largest tropical wetland area. Most of this biome's floodplains are submerged on the wet seasons, where an extremely diverse number of aquatic plants can be found [8].

In spite of its notable biodiversity, only in the last 15 years, microbial diversity studies in natural Brazilian environments have become more abundant [9–20]. However, these studies focused mostly on bacteria and fungi, with archaeal diversity being considerably less studied in comparison. The first report that described the presence of Archaea in Brazilian environments was a study focused on the microbial diversity of Amazon soils, published in 1997. This work described the finding of two crenarchaeal DNA sequences amongst 100 PCR products amplified from soil samples using prokaryotic universal primers, with the 98 remaining being classified as Bacteria [21].

**Figure 2:**
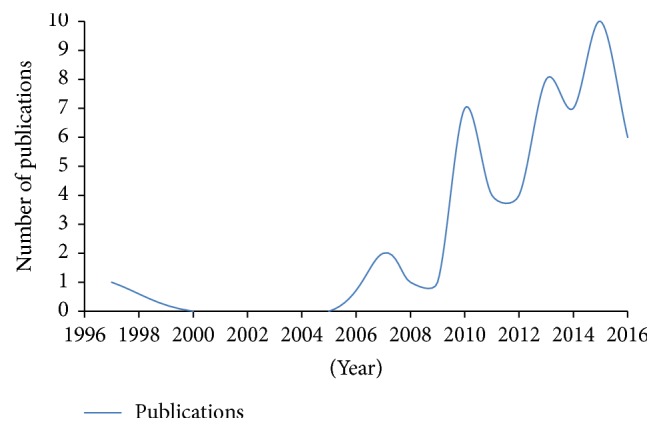
Number of published studies on archaeal communities in Brazilian environments.

Curiously, only in 2007, after a 10-year gap, the first study specifically focused on archaeal diversity in Brazilian environments was published [22]. Since then, the number of publications about archaeal communities in our biomes has increased, but in an irregular fashion. In order to better evaluate the progress of research groups involved in the description and characterization of Archaea in Brazilian natural and impacted environments, we have performed, in the last week of April 2016, a search on PubMed using the keywords “Archae^*∗*^ and Brazil^*∗*^” in the title and/or abstract fields. Using these criteria, and after the elimination of papers whose title or abstract were out of our scope, we were able to recover 51 publications, distributed from 1997 to 2016, as depicted on Figure 2.

Although our search intended to find all or the majority of publications, we are aware that probably some of them have not been included. For example, we could not retrieve a book chapter which describes the diversity of Archaea in Brazilian aquatic environments, published in 2011 [23], but which has been included here.

**Figure 3:**
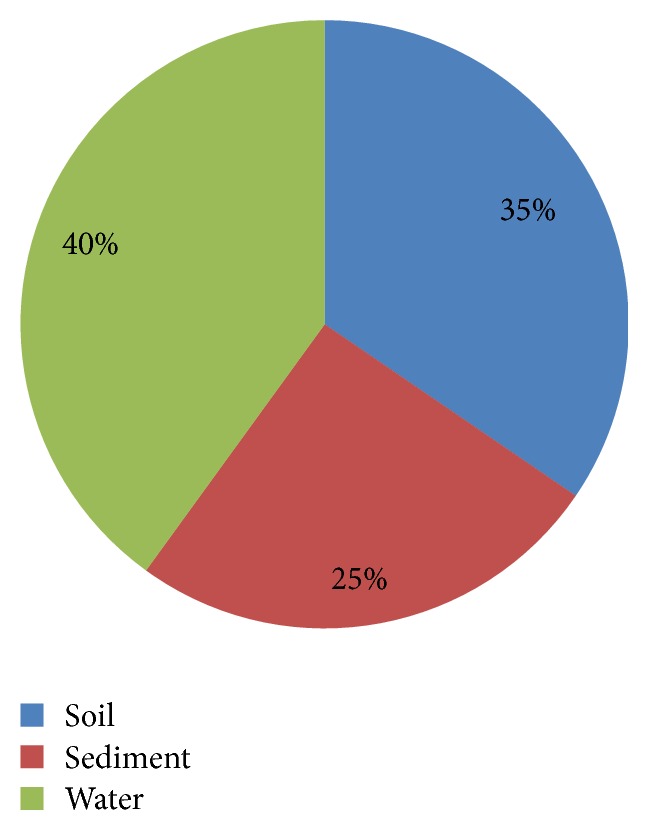
Distribution of the studies found in this survey according to the type of environment analyzed.

The environments studied in those publications were divided in the following categories: water, soils, and sediments, with a predominance of researches focusing on water environments, followed by soils and lastly sediments (Figure 3). Thus, this review will describe the major results and conclusions obtained in each of these environments.

## 2. Archaea in Aquatic Environments

As mentioned before, the first work focused on archaeal diversity in Brazilian environments was published in 2007. In this pioneering study, the authors compared the archaeal diversity among superficial water samples collected from sites with different pollution grades in the Guanabara Bay, an eutrophic estuarine system. According to their results, the water samples collected from polluted areas presented higher archaeal diversity when compared to pristine waters. Curiously, almost no crenarchaeotic DNA sequences were found, with a great abundance of sequences belonging to methanogenic and marine euryarchaeotic groups [22]. Since then, numerous works have been published describing archaeal communities in coastal waters and freshwater of natural or impacted areas, as well as in marine invertebrates.

Coastal waters were studied in two regions of Brazil, Arraial do Cabo and Ilha Grande, in the southeast region [24–27] and in the St. Peter and St. Paul Archipelago, in the northeast [28]. These studies analyzed the influences of upwelling and downwelling, as well as anthropogenic activities on the microbial/archaeal communities, since in all studies water samples were collected from pristine and sewage disposal subjected areas. Globally, the authors found that in areas subjected to anthropogenic activities the microbial richness was lower than in natural or conserved areas. The archaeal diversity was higher in samples collected from the surface, when compared to those obtained at higher depths. The first reports classified the archaeal DNA sequences obtained only as euryarchaeotes and crenarchaeotes, with no further additional classification. In some cases, the results revealed a slight predominance of the Crenarchaeotic Marine Group I [24], while in a more recent paper, the authors described the occurrence of DNA sequences associated to Euryarchaeotic Groups II and III, as well as others closely associated with* Cenarchaeum symbiosum* and* Nitrosopumilus maritimus* [25].

The influence of upwelling and downwelling in marine microbial communities revealed that in the upwelling area nitrate and phosphorus concentrations increased, while ammonium concentration decreased, with a discrete reduction of bacteria and archaea abundances, but an increase in* Nitrosopumilus maritimus* numbers [27]. In another work, by using DGGE, the same research group found that the archaeal communities were more homogeneous than the bacterial ones in water samples of sites with different degrees of upwelling and downwelling, as well as anthropogenic activities. The archaeal DNA sequences found were clustered in two groups: one associated with coastal waters archaea, and the other with open-sea archaeal DNA [26]. Taken together, the results obtained in these coastal waters studies revealed no significant differences in the archaeal communities of natural and polluted areas.

The study made in the northeastern archipelago of St. Peter and St. Paul employed pyrosequencing of water and coral samples and revealed that, among nearly 450,000 sequences, 0.4% corresponded to Archaea, with 58% belonging to the phylum Euryarchaeota and 21% to Thaumarchaeota. The specific abundance of the groups varied according to the nutrient concentration and turbulence, with Euryarchaeota being more abundant when the turbulence and nutrients were lower and Thaumarchaeota predominating when nutrients and turbulence were higher [28]. These results are in accordance with the findings of Euryarchaeota predominance in superficial waters and Thaumarchaeota in deeper waters [29, 30].

Archaeal communities were also studied in sponges and corals of protected and unprotected marine areas [28, 31–34]. In healthy* Siderastrea stellata* corals and different sponges, the archaeal diversity was higher, but the majority of DNA sequences were classified as uncharacterized crenarchaeotes, while in* Mussismilia hispida*, an endangered coral species, and also in some sponge species, sequences related to* N. maritimus* and* C. symbiosum* were detected. These findings are in accordance with works performed with Australian corals [31, 32]. On the other hand, corals reefs from unprotected areas revealed a lower diversity and abundance of Archaea than that of the healthier ones [33, 34]. Complementing those data, a more recent work, employing pyrosequencing revealed a broader distribution of Archaea in corals [28], describing the presence of Euryarchaeota (45.61%), Thaumarchaeota (42.11%), and Crenarchaeota (10.96%). A metagenomic study of rhodoliths that form the largest CaCO_3_ bed in the South Atlantic Ocean revealed that Thaumarchaeota was the dominant archaeal phylum in this kind of environment [35], again reinforcing previous findings of its higher abundance in deeper marine waters.

In spite of their importance, archaeal communities in heavily impacted environments have been poorly studied in Brazil, with only three articles retrieved in our search. The first described archaeal DNA sequences found in acidic waters were from a zinc mine that was discharged in Sepetiba Bay, in Rio de Janeiro. The authors have collected samples from sites with varying degrees of chemicals contamination, inside and outside of the mine, from a lagoon where the waters used to be discharged, and from the Bay. Their results showed that, except for the sample corresponding to the Bay water, all sequences were classified, by the RDP (Ribosomal Database Project), as uncultured crenarchaeotes affiliated to sequences retrieved from extreme environments such as other mine drainages and volcanic sediments. Methanogenic archaea have been found only in the lagoon sample, while the sample obtained from Bay water was rich in marine archaea, as expected [36]. The second study has focused on the methanogenic community of a highly polluted estuarine environment, located near a huge harbor, in São Paulo, employing enrichment cultures and FISH, revealing the predominance of Methanobacteriaceae and* Methanosarcina* spp. [37]. The third article described the phylogenetic analysis of the microbial community in hypersaline petroleum produced water from the Campos Basin, in Rio de Janeiro. Interestingly, the results suggest that this kind of environment is highly specialized in terms of the archaeal community, since most of the sequences (97.5%) were affiliated to Methanomicrobia, indicating that the basin waters are dominated by acetotrophic methanogens, with a smaller fraction of the hydrogenotrophic ones [38].

Extreme aquatic natural environments have also been poorly studied until now, restricted to two articles that describe the archaeal community in a hypersaline lagoon or hypersaline sediment. As expected, the authors found a great dominance of halophilic euryarchaeotes in their samples and a small number of crenarchaeotes associated with lineages found in marine vents. Curiously, in one of the samples, the halophilic sequences formed a distinct clade, suggesting the presence of new, undescribed, organisms in these waters [39, 40].

While most of the research performed in marine environments has been done in the southeast and northeast regions, the articles describing freshwater Archaea communities were made mainly in the Amazon region (located in the north region of Brazil) and in states located in the south region, such as Paraná and Rio Grande do Sul [41–46]. One exception is one study that described the freshwater archaeal community found in Ilha Grande, Rio de Janeiro, where the phylogenetic tree revealed that the DNA sequences obtained were affiliated to the Thaumarchaeota clade, especially to* N. maritimus* [25].

The microbial diversity of a hydroelectric power station reservoir in Brazilian Amazonia was studied, with sampling of waters from the photic and aphotic zones, as well as from the sediment of the reservoir. Detection of euryarchaeotes was possible and although some of the sequences were classified as Thermoprotei, differences in database version may lead to uncertain archaeal taxonomic affiliation. Globally the authors described that their samples presented 30% of Methanomicrobia and 40% of Thermoprotei. The photic zone revealed a higher proportion of euryarchaeotes and crenarchaeotes, while the aphotic zone was rich in methanogenic and halophilic archaea [41].

The Amazon River microbial communities were studied by metagenomics and metatranscriptomics, with Archaea representing 6% of the 16S rDNA sequences found in pristine waters. Among these sequences, those more similar to* N. maritimus* and* C. symbiosum* were predominant [42]. In the metatranscriptomic study, archaeal sequences corresponded to 3% of the genes identified and 10% of the total transcripts obtained. Since this work was focused on the inventory of the lower Amazon River, the authors have not discussed what types of Archaea were found and their ecological importance in this environment [45].

Two studies by the same group described the seasonal dynamics, community structure, and the distribution of microbial communities in upper Paraná River floodplain. The authors have studied Bacteria and Archaea in this environment, correlating the pulses of organic matter with the community variations when the river waters were high or low, and concluded that Archaea was less abundant than Bacteria in all times and seasons sampled [43, 44].

A very interesting and robust study describing the source of the river as a nursery for microbial diversity was published in 2015 using River Sinos as a model to evaluate the microbial profile along the river course and its relation to seasonal and environmental changes [46]. This river, located in an important industrial center in the south of Brazil, was chosen because it can be subdivided in three sections, which differ in terms of water flux and anthropogenic impacts. By high-throughput DNA sequencing techniques, a total of 5.790.065 reads were retrieved, with 1.777 belonging to the Archaea domain, which were grouped in 85 OTUs classified as Parvarchaeota, Crenarchaeota, and Euryarchaeota (curiously, the authors have not considered Thaumarchaeota as a phylum). Their results indicated a crescent predominance of euryarchaeotes from the higher to the lower river in the summer, with an opposite tendency in the winter.

Globally, the results described in this review are in accordance with the data presented in an elegant review describing two decades of studies focusing on microbial diversity in aquatic environments [47]. The authors showed that in spite of the increasing interest in this kind of environment, the number of publications and kind of sampled area describing Archaea are still restricted when compared to Bacteria, which represented 77.6% of the selected literature in their review. As observed by these authors, we also verified that, in Brazil, the most studied aquatic environment corresponded to saline and mixed saline waters, with a smaller number of publications describing freshwater and impacted aquatic communities.

After the first detection of Archaea in water bodies of nonextreme environments [48, 49], most of the studies done so far focused on archaeal phylogenetics and not on their ecological roles in these environments. After the discovery of ammonia oxidizing archaea (AOA) by Könneke et al. [50], a great number of papers have been published describing the detection of this group in natural and impacted aquatic environments around the world, trying to establish their potential roles in nitrogen cycling [51–60]. Curiously, the results are controversial with respect to the abundance and importance of AOA and AOB (ammonia oxidizing bacteria) in water bodies.

## 3. Archaea in Marine and Freshwater Sediments

Ever since studies on archaeal diversity in Brazilian environments became more abundant, different types of sediments have been investigated. There are studies on mangrove, lake, stream, and marine sediments, which were conducted in a variety of Brazilian biomes. One of the earliest reports investigated oceanic sediments in Rio de Janeiro [39]. This study used 16S rDNA libraries and found that most of the sequences were related to Euryarchaeota halophilic archaea, though a small number clustered in a group of hydrothermal marine sediment crenarchaeotal sequences.

Other studies investigated archaeal diversity in mangrove sediments. A 2010 study by Taketani et al. [61] analyzed sediment samples from a mangrove on Cardoso Island, located in São Paulo. In this study, methanogenic archaea and sulfate reducing bacteria diversity were investigated in depths up to 50 cm using DGGE-PCR and DNA libraries (*mcrA* gene). Results revealed a decrease in archaeal richness as sediment depth increased, with most sequences clustering in clades affiliated to* Methanopyrus*,* Methanothermococcus,* and* Methanosarcina*. The authors discussed that sulfate reduction and methanogenesis are both responsible for organic matter electron removal in anaerobic environments and that these processes are commonly exclusive in environments where electron acceptor availability plays an important ecological role. However, a cooccurrence of sulfate reducing bacteria and methanogens was detected, indicating either that the abundance of organic matter allows the presence of these organisms by reducing substrate competition or that the detected methanogens are capable of using substrates that are not used by sulfate reducing bacteria. A recent article described the dynamics of hydrogen transfer between an acetoclastic methanogen and hydrogenotrophic microorganisms, grown in pure and mixed cultures, and the results suggest that these organisms coexist and do not compete in marine sediments [62].

A later study of the same research group [63] investigated archaeal communities on the same sampling site, Cardoso Island, focusing on nonmethanogenic Archaea. According to the results obtained, besides the physicochemical parameters, such as pH, organic matter, potassium, and magnesium, depth was also a factor that influenced the archaeal community composition. In samples collected at 50 cm, members of Crenarchaeota and Thaumarchaeota represented 75.7% of all sequences found, while euryarchaeotal sequences corresponded to 24.3%. However, the opposite was observed as depth decreased, with sequences affiliated to Euryarchaeota representing 74.3% of the sequences retrieved in these conditions.

Archaeal communities in mangrove sediment rhizospheres from the Guanabara Bay in Rio de Janeiro were investigated to evaluate the influence of mangrove plants in the microbial community [64]. The rhizospheres of* Rhizophora mangle* and* Laguncularia racemosa* were studied and significant differences between the archaeal community in the sediment and the rhizosphere were detected. The archaeal community was the richest on the* L. racemosa* rhizosphere, followed by the sediment community and then* R. mangle* rhizosphere. Interestingly, members of Methanomicrobia were detected in the rhizosphere communities but not in the sediment, suggesting that the conditions close to the plant's roots favors methanogenesis due the production of reduced compounds in these environments. The study of mangrove sediments of Ilha Grande, in Rio de Janeiro, revealed DNA sequences belonging to the Thaumarchaeota phylum, related to* N. maritimus*. Sequences that clustered with uncultured organisms, commonly referred to as the Miscellaneous Crenarchaeotic Group (MCG), were also detected along with sequences affiliated to Group III and LDS/RCV Euryarchaeota [25].

Lake sediments were also described in three studies. One of them investigated methanogenic microbial communities in Amazonian oxbow lake sediments during desiccation stress. Sediments were incubated in anoxic conditions, desiccated, and then rewetted. Methane production was detected in all experimental conditions, with higher rates in the later stages. Quantitative PCR assays revealed that the number of archaeal rRNA 16S gene copies in the desiccation and rewetting processes varied among sediment samples. There were samples in which archaeal 16S rRNA gene copies decreased only after rewetting, while in others the number decreased with desiccation but increased after rewetting. Interestingly, qPCR using the* mcrA* gene followed the same pattern observed with the 16S rRNA gene. Up to 85% of archaea detected were methanogens, with the remaining classified as Crenarchaeota. In some samples* Methanocella* increased in relative abundance after the processes, while in others there was an increase of* Methanosarcina* [65]. These results are in accordance with other studies describing the relative good resistance of methanogens to oxic desiccation [66] and their enrichment in oxic soils [67].

Lake sediments of Cerrado biome collected in the dry season and in the transition between the dry and rainy seasons were investigated by our group. In this study, methanogens and MCG were predominantly observed in the dry season, while members of Thaumarchaeota were dominant on the transition period, which was characterized as less diverse than the community found in the dry season. Since water flow originated by rains promotes resuspension and redeposition of the sediment surface, leading to changes in the sediment nutritional composition, we suggested that the increase in nitrogen compounds observed after the occurrence of rain could have inhibited the growth of methanogens, favoring ammonia oxidizing archaea (AOA) [68].

The microbial distribution through space and an evaluation of biogeographic patterns occurrence were described in sediments of “Lagoa Negra,” a lagoon located in Pantanal. Twenty-six sediment samples were submitted to T-RFLP analyses directed to archaeal 16S rRNA and* mcrA* genes. The *z* value found for the increase in the number of species in relation to distance was lower than in other studies, indicating a flat species-area correlation. However, distance-decay slopes were calculated and all results were considered significant, with* mcrA* genes having the highest slope [69].

The diversity of archaea in natural and impacted streams has been studied in the State of Minas Gerais. One study [70] investigated the effects of arsenic, commonly associated with mining activities, on the microbial community of stream sediments from the Iron Quadrangle of the Velhas River Basin. The authors found higher archaeal diversity in impacted sediments when compared to sediments from a stream not influenced by mining activities. In arsenic impacted sediments, Crenarchaeota represented 75% of the sequences obtained, with the remaining 25% classified as Euryarchaeota. In the nonimpacted samples, community composition was notoriously different, with Euryarchaeota representing 58% of the sequences and Crenarchaeota 42%. The euryarchaeotal OTUs were associated with* Methanosaeta *sp. and* Methanosarcina *sp., suggesting that both acetate and other compounds such as methanol and methylamines were being used as substrate for methanogenesis. A subsequent study by the same group [71] investigated the effect of human settlement in mining areas on ammonia oxidizing microorganisms present in these kinds of sediments. Archaeal* amoA* genes were detected in both impacted and nonimpacted sediments, but most abundantly in the nonimpacted sediment. On the other hand, bacterial* amoA* genes were detected in higher numbers in impacted sediments.

Recently, a metagenomic study conducted in the same area described the functional composition of microbial communities on metal contaminated stream sediments. Taxonomic analyses using the rRNA 16S gene as marker revealed that most of the OTUs found were from the Parvarchaeota phylum. Although no thaumarchaeotes were detected in these analyses, it was possible to partially assemble the genome of three thaumarchaeotal species:* Nitrosopumilus maritimus*,* Cenarchaeum symbiosum,* and* Nitrososphaera gargensis*. The group proposed a putative role of* N. maritimus* in the process of nitrogen cycling in these sediments. The functional reconstruction of the sediment's metagenome revealed the presence of diverse gene sets related to ammonium assimilation as well as a high diversity of metal resistance genes, which is coherent to the sediment samples used in the study [72].

Oceanic sediments from harbors in Ceará state, on the northeast of Brazil, were also investigated by metagenomics and DGGE [73]. Superficial sediments were retrieved in two port areas, one isolated and less affected by human activities (Pecém port) and the other located on a polluted metropolitan area (Mucuripe port). DGGE analyses revealed a more homogenous microbial community in the polluted port. Among the 470.000 sequences obtained in metagenomic analyses, 3% were classified as Archaea. On Pecém port, Thaumarchaeota was the dominant phylum, representing 95% of the archaeal sequences, while on Mucuripe port most sequences were assigned as unclassified Archaea.

The finding of a great abundance of methanogens on sediments of different Brazilian biomes is coherent to what has been extensively described in the literature [74–78], given the anoxic conditions and high organic matter amounts commonly found in this type of environment [79]. In recent years members of the group formerly known as Miscellaneous Crenarchaeotic Group, MCG, now proposed as phylum Bathyarchaeota [80], has been investigated in the sea subfloor and in marine sediments, revealing their potential importance in methane and nitrogen cycling [81–84]. In spite of its huge coastal area, to our knowledge, this kind of study has never been performed in Brazil.

## 4. Archaea in Soils

Members of Archaea are widely distributed in soils all over the planet, with a great number of studies focusing on their diversity in this terrestrial environment around the world [85–89]. As mentioned before, the presence of Archaea in Brazilian environments was described for the first time in a study focused on the microbial diversity of Amazon soils, published in 1997 [21].

Although Amazon soils are generally described as acidic and with low fertility, Anthrosols (World Reference Base classification), which are fertile dark soils, rich in organic matter, with carbon in the form of biochar, can also be found in the Amazon biome, where they are known as Terra Preta [90]. These are classified as man-made soils from ancient civilizations and are frequently found in forest vegetation but are not comprised of the general classification, which has Ferralsols and Acrisols (WRB classification) as the most frequent in the Amazon biome [91]. DGGE analyses of these soils showed a less diverse community of Archaea when compared to that of Bacteria. The banding pattern of Archaea revealed 90% of similarity between the Terra Preta and adjacent Oxisol soil, despite their great differences in edaphic characteristics. The ten sequenced DGGE clones were classified by the authors as Crenarchaeota, possibly linked to the accumulation of nitrate in those soils [92]. In another study comparing Terra Preta and an adjacent low-fertility soil, T-RFLP fingerprints and the 16S rDNA library analysis for the archaeal community showed no significant differences between the Terra Preta and the adjacent soils, probably due to other effects from the plantations in each site [93]. The adjacent soils presented higher diversity but greater redundancy, represented in 90% by* Nitrososphaera* and Candidatus Nitrosocaldus, while in Terra Preta these genera represented only 60% of the community. Both soils shared the most abundant OTUs, which was also true for the community of AOA [93].

This higher archaeal diversity detected in the adjacent soil in comparison with Terra Preta is opposed to the findings of Kim et al. [94] that have shown a greater bacterial diversity in Terra Preta soils. Similar results were observed in Cerrado biome, where soils from a riverine forest site showed greater bacterial diversity than the one found in soils from a shrubland site [17], while archaeal diversity was higher in the latter [95], suggesting that Archaea may prefer low-energy niches [96].

As mentioned in the introduction, Cerrado biome has a gradient of vegetation cover and can be divided into different phytophysiognomies that range from grasslands (Campo Limpo and Campo Sujo) to savannas (Cerrado* sensu stricto* and Cerrado Denso) and riverine forests (Mata de Galeria) [97]. Similarly to the Amazon biome, Cerrado soils are almost 50% classified as Ferralsols (WRB; Latossolos in the Brazilian Classification) [98]. Archaeal communities in soils from Cerrado biome were investigated for the first time by our group, in a study that described and compared archaeal diversity in native soils from two phytophysiognomies, Cerrado Denso and Mata de Galeria. All rRNA 16S gene sequences were affiliated to Thaumarchaeota, with a prevalence of thaumarchaeal I.1b subgroup in Cerrado Denso and I.1c subgroup in Mata de Galeria. Sequences affiliated to the Thaumarchaeota I.1a subgroup were only detected in Mata de Galeria soils, which was attributed to the higher water content of these kinds of soils. As mentioned, differences in archaeal richness and diversity were also observed, with higher values found in Cerrado Denso [95].

In a subsequent work, we investigated archaeal communities in typical Cerrado* sensu stricto* soils, which also revealed a predominance of Thaumarchaeota (data not shown). Soils from a typical Cerrado* sensu stricto* area protected from fires and from a similar area but submitted to a biennial fire regimen were analyzed and revealed higher archaeal richness in the protected area, in addition to higher relative abundance of I.1c thaumarchaeal affiliated sequences. On the other hand, soils from the frequently burned area had higher proportions of I.1b thaumarchaeotes and more* amoA* gene OTUs, indicating higher richness of AOA associated with this condition. Changes in vegetation structure and composition caused by periodic fires in the Cerrado biome could lead to long-term alterations on archaeal communities in soils (data not shown).

Recently, high-throughput sequencing of ribosomal markers and shotgun metagenomic analyses were combined to investigate bacterial, fungal, and archaeal communities in soils of four different phytophysiognomies from Cerrado during the dry and rainy seasons [99]. Most of the archaeal sequences detected in soils from three vegetation physiognomies (Campo Sujo, Cerrado* sensu stricto* and Mata de Galeria) in both seasons were affiliated to Crenarchaeota and classified as Thermoprotei. It is known that Thermoprotei is a class of thermophilic or hyperthermophilic organisms and are unlikely to predominate in archaeal communities of mesophilic soils such as the ones found in Cerrado regions. Additionally, it is also known that taxonomic classifications made exclusively by database comparisons can result in discrepancies, especially when different versions of databases are employed [100]. Thus, probably some of the sequences classified as Thermoprotei are actually affiliated to lineages that are increasingly detected in mesophilic environments but that still have unstable taxonomic classifications. The phylum Thaumarchaeota was the most abundant in soils from Cerrado Denso in both rainy and dry seasons and although sequences affiliated to Methanomicrobia were found in this phytophysiognomy during the rainy season, they were absent in the dry season samples [99].

The archaeal diversity in peatland soils from the Atlantic Forest has been also analyzed and, in contrast to what has been observed for peatland soils of the northern hemisphere, methanogens were not dominant in the communities from the sampled soils [101]. In the Brazilian study, 90% of the sequences detected belonged to the MCG and the Crenarchaeotic Terrestrial Group (probably a Thaumarchaeota group). Interestingly, three MCG sequences detected in typical Cerrado* sensu stricto* soils were closely affiliated to sequences detected in these Atlantic Forest soils (data not shown). Depth seemed to influence the archaeal distribution in Atlantic Forest peatland soils, since communities from deeper soils (50 cm) showed lower percentage of exclusive OTUs than observed in shallower soils (20 cm). Methanogens represented 9% of all obtained archaeal sequences, with the Rice Cluster-II (RC-II) being the most abundant group and sequences related to the* Methanosaeta *and* Methanoregula* and* Methanocella* genera were also detected. Additionally, in the sampling sites where sequences related to* Methanosaeta* were found, no sequences of Methanosarcinaceae were detected, suggesting low concentrations of acetate in this peatland, since in ecosystems with high acetate concentrations* Methanosaeta* are outcompeted by* Methanosarcina* [101].

Different horizons of three podzol profiles located in cliffs formed by marine erosion along the coastline in São Paulo were also analyzed for archaeal communities. PCR-DGGE analyses and sequencing of rRNA 16S gene fragments revealed that Archaea were mostly affected by soil depth, with the community structure in soil samples from the upper horizons more similar to each other than to the community structures in the deeper horizons in all samples sites. Furthermore, soil areas affected by bleached mottles were compared with nonbleached adjacent soil from the same podzol profiles and no statistical differences in archaeal communities were observed in most soil profiles. Thus, the role of Archaea in tropical podzols remains unclear [102].

Influence of soil depth in archaeal communities reported in the Brazilian Atlantic Forest [101] and costal podzol profiles [102] was also described in other studies worldwide, with pronounced shifts in archaeal communities along depth gradients observed in soils from a Norway spruce-beech forest in Switzerland [103], a German mixed deciduous forest [104], and a continuous soil profile within the Melton Branch Watershed in the United States [105].

In another study, Lima-Perim et al. [106], using PCR-DGGE, qPCR, and both rRNA 16S and* amoA* genes pyrosequencing, described bacterial and archaeal communities along a natural altitudinal gradient in soils from an Atlantic Forest area. Among the sequences classified into known archaeal phyla, 51% were affiliated to Thaumarchaeota, 30% to Crenarchaeota, and 19% to Euryarchaeota, with a prevalence of thaumarchaeotal sequences at higher altitudes, while Crenarchaeota and Euryarchaeota were more frequently found at the sea level. Archaeal richness and diversity indexes were similar among all sampling sites and although bacterial 16S rRNA genes were more abundant in all sampled soils, archaeal* amoA* genes were more abundant than the bacterial ones in all conditions, which could be linked to the low pH of sampled soils. Both archaeal and bacterial* amoA* genes were less abundant at the sea level soils and it was suggested that rain characteristics at lower altitudes may lead to lower oxygen availability in Atlantic Forest soils, impairing ammonia oxidation based metabolism. pH is known to be a driver for bacterial diversity [107] and specifically for AOA [108], but also for archaeal communities in general, as highlighted recently in a comparison between tropical and temperate biomes, as Archaea and specifically AOA have a negative correlation with pH [109].

A recent metagenomic study detected Thaumarchaeota, unclassified Crenarchaeota, and Halobacteria (Euryarchaeota) sequences in soils from an environmental conservation area of Atlantic Forest in northeastern Brazil, as well as in soils from a semiarid area of Caatinga, another Brazilian biome [110].

The studies referred above describe Archaea in native environments in Brazil. Nevertheless, others have evaluated the impact of modified environments, as agriculture or petroleum-contaminated soils, over the archaeal community. The effect of distinct soil types and land-uses on the structure of archaeal and fungal communities in soils from the Pampa biome was investigated by RISA and it was seen that both of these two conditions had strong influence on these microbial communities. However, archaeal communities were mainly affected by soil type, while fungal communities were mostly associated with land-use changes [111]. Others suggested that fungi and bacteria seemed to be suppressed by pesticides in soybean fields, while there was an increase in archaeal abundance in old soybean sites, possibly affected by other factors as plant cover or tillage [112].

Archaeal communities of native forest and of oil palm plantation were evaluated in the Amazon biome and showed a smaller number of OTUs in the oil palm cultivation area than in the native forest. In addition, a greater abundance of Euryarchaeota (Thermoplasmata, 21%) was detected in the former, while Thaumarchaeota (Soil Crenarchaeotic Group, 26%) relative abundance was greater in the native sites [113]. There was a separation of OTUs between the two sites, corresponding to either I.1b or I.1c thaumarchaeotic groups, with the remaining clusters affiliated to MCG. Methanomicrobia abundance was increased in the oil palm site but also represented in the native forest soil. On the other hand, the detection of Methanomicrobiales exclusively in the native forest sites was explained by the higher levels of carbon and organic matter found in those soils. Interestingly, there was also a clear distinction of abundance of two AOA between the two sites sampled:* Nitrososphaera* predominated in the native forest soil (18.2% of the sequences, against 10.3% of the oil palm site) and* Candidatus* Nitrosotalea in the oil palm cultivated site (10%, against 4.4% in the native forest). The authors suggested that this differentiation could be due to the addition of N fertilizers in the oil palm field, leading to an increased abundance of* Candidatus* Nitrosotalea. The detection of those AOA in both sites was discussed to be related to the acidic pH in those soils [113].

As observed for the 16S rRNA gene, AOA were more abundant than their bacterial counterparts (AOB, bacterial ammonia oxidizers) in all sites but especially in the old soybean sites compared to pasture and native forest sites within the Amazon biome. A comparison of functional guilds for the N cycle between pasture and soybean plantation soils within the Amazon showed greater relative abundance of Archaea in the soybean site than in the pasture or the native forest site. Archaeal abundance was negatively correlated with C content in soils, and AOA (and AOB) was significantly correlated with NO_3_^−^ concentration [112]. In the Cerrado biome, AOA was also detected in higher abundance than AOB in a soybean plantation; however, AOB increased in abundance between the beginning of the site management for agriculture and the soybean blossom stage, while AOA abundance did not change [114].

In most descriptions of terrestrial environments, only the bulk soil is evaluated. Recently, a study showed differences among the rhizosphere microbial communities, but not for the archaeal community specifically, in coffee plantation (*Coffea arabica* L) farms, located in the southeastern part of Minas Gerais State, with different soil management: organic (for 4 and for 18 years) or conventional management with pesticides and fertilizers [115]. The archaeal sequences were classified as Thaumarchaeota (89.7%), Euryarchaeota (8.3%), and Parvarchaeota (1.9%) and in both intensive and organic managment soils, the genus* Nitrososphaera* corresponded to the dominant organism. The highest amount of* Nitrososphaera* was found in the intensive management, which was negatively correlated with the abundance of* Bradyrhizobium* [115].

Another study on the rhizosphere soil focused on leguminous plants (*Mimosa tenuiflora *and* Piptadenia stipulacea)* in two seasons (wet and dry) from a semiarid biome named Caatinga, in the northeast of Brazil, with low incidence of rain (500–800 mm year^−1^). However, it was not possible to detect any significant difference in the archaeal community for the two plants, neither in relation to the different points nor in relation to seasons [116].

Sedimentary soils of a petroliferous basin in northeast Brazil were evaluated for archaeal and bacterial communities by rRNA 16S gene libraries. Although chromatographic data revealed relatively high concentrations of methane in these soil samples, only 2% of all archaeal sequences were affiliated to the methanogenic organism* Methanosarcina acetivorans, *while 56% of all archaeal sequences detected were classified as uncultivated Crenarchaeota and 42% were affiliated to the Thaumarchaeota* Candidatus* Nitrososphaera gargensis. As observed for other soils [99, 117], archaeal richness and diversity values were significantly lower than those found for bacteria in the sedimentary soils from this petroliferous basin [118]. In another study, the effect of petroleum contamination on microbial communities from Trindade Island coastal soils were analyzed by microcosm experiments [119]. High-throughput sequencing was performed to compare bacterial, archaeal, and fungal rRNA 16S genes in microcosms assembled with soil contaminated with crude oil and noncontaminated soils. A significant reduction in diversity of the three domains was observed upon oil addition. In noncontaminated soils, archaeal sequences represented 6% of all sequences and the addition of oil reduced archaeal sequences relative abundance to 2.7%. Archaeal taxonomic classification revealed sequences affiliated to the genus* Nitrososphaera*, to the Euryarchaeota order E2, and to the Parvarchaeota phylum. The ammonia oxidizing archaea* Nitrososphaera *sp. represented 6.1% of total sequences in noncontaminated soils and only 2.8% in petroleum-contaminated soils, a reduction that was attributed by the authors to the higher sensitivity of AOA in comparison to AOB to crude oils hydrocarbons, observed in other studies [119, 120].

Interestingly, these results suggesting a reduction of archaeal relative abundance in oil-contaminated soils from Trindade island are controversial to what was observed for Chinese oilfields contaminated soils, which detected an increase of archaeal sequences [121]. In this study, soils from six major oilfields in China were compared with undisturbed soils and the number of archaeal sequences, mostly associated to halophilic Euryarchaeota, was much higher in contaminated soils, suggesting that oil contamination may stimulate the enrichment of such archaea and indicating that, as proposed by other studies [122–124], halophilic archaea may have a role in oil biodegradation in certain* in situ* geochemical conditions.

In the past years, many researches have focused on archaeal communities in soil, perhaps making it one of the most studied environments worldwide. Globally, as observed in Brazilian soils, Archaea in dry soils appear to be predominantly Thaumarchaeota (previously classified as Group I Crenarchaeota, with some databases and studies still considering this nomenclature), with the thaumarchaeal I.1b group being reported as dominant in different soil types from a broad variety of ecosystems, ranging from grasslands and semiarid shrublands to temperate and tropical forests [89, 125–129]. Group I.1c Thaumarchaeota are commonly found in boreal forests, grasslands rich in organic matter, and different acidic soils [86, 125, 128, 130], while Group I.1a Thaumarchaeota has been detected in Russian grasslands [125] and agricultural soils [131], although it appears to be more rare in terrestrial habitats than Groups I.1b and I.1c.

As previously mentioned for aquatic environments, since the first description of an ammonia oxidizing archaea [50] and also due to the great occurrence of groups involved in this metabolism in soils (specially Group I.1b Thaumarchaea), AOA abundance and diversity have been intensively researched in terrestrial habitats submitted to different conditions [88, 132–136]. Furthermore, the abundance and specific contributions of AOA and AOB in soils have been increasingly investigated, with AOA apparently dominating in some types of soil and specific conditions [137, 138], while AOB apparently predominate in others [139, 140].

Although Bathyarchaeota (MCG) is mostly associated with marine and freshwater sediments, they have also been detected in a variety of soils worldwide [81, 141, 142]. Thus, since this group appear to have an unstable taxonomic classification in phylogenetic trees based on rRNA 16S [81], sequences sometimes classified as Crenarchaeota could actually be affiliated to Bathyarchaeota. Furthermore, Archaea affiliated to the Euryarchaeota phylum have also been detected in different kinds of soils, although less frequently and with lower diversity in dry oxic soils worldwide [103, 128, 143–146].

## 5. Concluding Remarks

Surprisingly, given the size and biome diversity of Brazil, the number of publications describing archaeal diversity and ecology is proportionally small, since our survey has retrieved only 51 articles, in the last 20 years. Although the first study was published in 1997, only after 2007 publications have become more frequent but, since then, in an irregular fashion. This could be explained by the low number of research groups directly involved in the study of Archaea in Brazil. Additionally, a significant number of publications discussed here were focused on microbial diversity of natural and/or impacted Brazilian environments, and not on archaeal diversity. In this context, it is understandable that, given their scope, the authors have provided a less detailed discussion regarding archaeal diversity.

Another aspect that must be considered in a review of this kind is the fact that in the last years the taxonomy of archaea has become more complex, with many new phyla being proposed in the last years. When the Archaea domain was proposed, in 1990, the authors already described the existence of two major archaeal lineages, the Crenarchaeota and Euryarchaeota phyla [147]. However, a review of the recent literature with respect to the archaeal systematics reveals that in the last years the phylogenetic tree of Archaea was subjected to profound changes, with new phyla and superphyla proposed, as well as the description of new archaeal organisms that are being considered as the putative links between the Archaea and Eukarya domains.

Advances in molecular techniques and the increase in culture-independent studies focusing on archaeal diversity from a broad variety of environments led to an expansion of our knowledge about the Archaea domain. The phylum Korarchaeota was proposed after analyses of 16S rRNA gene sequences from samples retrieved from the Obsidian Pool, in the Yellowstone National Park, USA, which was classified to a basal lineage in the archaeal tree [148]. The phylum Nanoarchaeota was proposed in 2002, after the isolation and cultivation of a small archaeal cell, which grows as an exosymbiont of the Crenarchaeota* Ignicoccus hospitalis* [149]. By using other evolutionary markers, such as ribosomal proteins, in 2008 the phylum Crenarchaeota was subdivided, with the proposal of the phylum Thaumarchaeota, which included all AOA described so far [150]. Furthermore, high-throughput sequencing methodologies, combined with metagenomic reconstructions and single-cell analyses, allowed the detection and proposal of many other phyla in the last five years, including Aigarchaeota, Geoarchaeota, Parvarchaeota, Aenigmarchaeota, Diapherotrites, Nanohaloarchaeota, Bathyarchaeota, Woesearchaeota, Pacearchaeota, Lokiarchaeota, and Thorarchaeota [80, 151–156]. However, most of these new taxa have been proposed exclusively based on genetic data and although these findings truly help us to better understand archaeal phylogeny, the paucity of cultivated representatives of many of these new groups, as well as the lack of complete genome sequences, still hamper their true importance and position in the phylogenetic tree.

Due to the constant updates in archaeal taxonomic classification and the rapid increase in the number of archaeal sequences deposited in databases, there is a lack of consensus with respect to their nomenclature. For example, although the Thaumarchaeota phylum has been proposed in 2008 [150], some authors still consider nitrifying archaea as crenarchaeotes. This is also true for the Bathyarchaeota, a recently proposed phylum [80]. Besides the taxonomical and nomenclature problems, it is also worth mentioning that comparisons among the studies discussed here were also hindered by methodological aspects, since a plethora of techniques, such as Next Generation Sequencing, PCR, qPCR, T-RFLP, RISA, and DGGE, were used. Furthermore, the use of different versions of multiple databases has to be mentioned.

Certainly, the studies retrieved here have a great importance in shedding a light on the archaeal ecological dynamics in natural and impacted environments of different Brazilian biomes. We can conclude that, despite the different samples used in the studies, water, sediment, or soil, the markers employed to characterize these organisms were the 16S rRNA,* mcrA,* or* amoA* genes. Taken together, the results indicate that Euryarchaeota was the phylum more commonly found in water and sediment samples, while Thaumarchaeota was more dominant in soil samples, with most of ecological discussions related to methanogenesis and ammonia oxidation. Interestingly, the metabolic pathways that were better described are those found in cultured isolates. In this sense, the importance of culturing new archaeal organisms to thoroughly understand this domain's biology is widely accepted, as there are many groups known only by 16S rRNA gene sequences. Although new microbial species have been described in Brazil in the last years, none of them were Archaea [157]. Thus, new cultured isolates as well as more environmental surveys will surely improve our knowledge on archaeal diversity and ecology in Brazil.
